# Canadian Federation of Medical Students (CFMS) response to: The unmatched by Dr. Amit Persad

**Published:** 2018-05-31

**Authors:** Kaylynn Purdy, Sarah Silverberg

**Affiliations:** 1 Northern Ontario School of Medicine, Ontario, Canada; 2 Faculty of Medicine, University of Toronto, Ontario, Canada

Many Canadian medical students fear that after years of rigorous training and personal and financial sacrifice they may be unable to secure a residency position - their only point of entry into becoming an independent practitioner in Canada. Our system is designed to produce some doctors who may be unable to work as doctors. It is a fear for thousands of medical students, and a reality for 114 medical students who were unmatched as of 2017 and 169 recently in 2018. The number of residency positions has not increased and therefore; the number of unmatched graduates is projected to climb should urgent action not be taken to address the current system.

During the residency application, interview offer, national interview periods, and ultimately the time leading up to “Match Day,” medical student stresses soar. Some students report having their first ever panic attack after submitting applications, others watch their dreams fade when they get a limited number of interview offers, and a few become paralyzed by anxiety during a high stakes interview. If a student makes it through all of these steps, they may be lucky enough to be told on Match Day, “We are pleased to inform you that you have been matched.” Others will have done everything right, been told they were good candidates, felt they did well in interviews, yet, come Match Day they receive: “We regret to inform you that you did not match into a position.” Truly, as in Persad’s essay,^1^ many, if not most of these students received no indication throughout their training that they were a poor student, an uncompetitive candidate, or received any other red flags to indicate they might not match.

In that moment, some students say the felt like their “dreams were taken away.” While the majority of medical students celebrate matching into a program of their choice, many students across the country dread what lies before them. Colleagues move on, with many moving to different cities for residency, going on dream vacations before starting their next phase of training, purchasing their first house, and settling into life as a resident. Unmatched students are stuck, repeating actions they had previously taken by seeking opportunities to strengthen residency applications, often by looking for opportunities to do further electives or research in their chosen field. Some may pay for an additional year of medical school, others pursue master’s programs, and some re-enter the workforce, all with the goal of strengthening their applications and hoping that the second time around they will be successful. A few students may leave clinical medicine all together. Their path once “unmatched” is unclear as well as daunting. There are many different options charted by only a few students unlucky enough to have gone before them with, unfortunately, varying levels of support from their home medical schools.

The reflections of Dr. Amit Persad^1^ on his journey as an unmatched student is an exceptional one, and does not reflect the norm. Dr. Persad did not match on his first try, however he was fortunate to secure an R1 residency position in his discipline of choice by July 1st of the same year - outside of the CaRMS R1 Match. This is a very rare set of circumstances for those who go unmatched. Unlike other systems around the world using an algorithmic match, there are few opportunities for Canadian students and programs to scramble to find opportunities for unmatched students. The vast majority of students must wait a year to enter the next R1 match, and struggle with financial hardship and mental challenges such as loneliness, low self-worth, lack of direction, depression, or hopelessness. While being unmatched represents a learning opportunity for some students, others struggle with the reality of having to re-consider new career trajectories in specialties they had not previously considered. Some medical students may enter the match cycle for two years before matching, or, in some cases, never match at all.

The realities of our postgraduate education system is that there are not enough residency positions with respect to medical school graduates. The recommended ratio for the algorithm to work best is 120 seats for every 100 students. In recent years, that ratio has approached 102 seats for every 100 students. When we look further into the data, it becomes clear that the ratio of anglophone applicants to anglophone positions in Canada is 0.986. This means that there will certainly be unmatched English speaking students each year, and likely some from every English language medical school graduating class in Canada. As the ratio of available positions to applicants drops, the math looks worse and worse. More water is being added to the pool every year, but the pool is not getting any bigger. It has now reached a point where our medical education system is overflowing with unmatched medical students.

Ambiguous admissions criteria for many residency programs further increase the anxiety that students face in submitting applications. Students believe that to be successful they must apply to more programs, more fields, and commit to areas of medicine earlier in their training. When students limit their clinical elective experiences to just a few specialties, it not only may compromise the education of future physicians, but, in combination with the opacity in selection criteria, such emphasis on a narrow career trajectory creates a heavy emotional burden for medical students to carry.

Medical students face the prospect that they can complete years of education, are qualified by all objective measures to continue training to be a physician, are many thousands of dollars in debt, and may never be able to practice medicine. This is not a situation that any person wants to face. Yet, this remains a frightening reality for many medical students, and with continued physician shortages, a reality with negative consequences for every Canadian.

We hope that continued efforts on the part of our medical educational leaders will help ease some of the emotional and financial burdens unmatched students face. The CFMS will continue to work to assist students to navigate the tumultuous waters that many must pass through in their pursuit of a fulfilling career as an MD. We hope that the light at the end of tunnel will continue to grow.
